# Quality of life in patients with chronic hepatitis C infection: Severe comorbidities and disease perception matter more than liver-disease stage

**DOI:** 10.1371/journal.pone.0215596

**Published:** 2019-05-03

**Authors:** Sabrina Cossais, Michaël Schwarzinger, Stanislas Pol, Hélène Fontaine, Dominique Larrey, Georges-Philippe Pageaux, Valérie Canva, Philippe Mathurin, Yazdan Yazdanpanah, Sylvie Deuffic-Burban

**Affiliations:** 1 Inserm, IAME, UMR 1137, Paris, France; Université Paris Nord, Sorbonne Paris Cité, Paris, France; 2 THEN (Translational Health Economics Network), Paris, France; 3 Unité hépatologie, Groupe Hospitalier Cochin Hôtel-Dieu, Paris, France; 4 Inserm U1223, Institut Pasteur; Université Paris Descartes, Paris, France; 5 Service des maladies de l’appareil digestif, Hôpital Saint Eloi, IBR- Inserm Montpellier, France; 6 Service des Maladies de l'Appareil digestif et de la Nutrition, Hôpital Huriez, Lille, France; 7 Université Lille, Inserm, CHU Lille, U995—LIRIC—Lille Inflammation Research International Center, Lille, France; 8 Service des Maladies Infectieuses et Tropicales, Hôpital Bichat Claude Bernard, Paris, France; Bielefeld University, GERMANY

## Abstract

**Background and aims:**

This study evaluated the clinical and non-clinical determinants of health-related quality of life (HRQoL) associated with untreated chronic hepatitis C (CHC) in France.

**Methods:**

From 01/2014 to 01/2015, untreated CHC patients were invited to complete a questionnaire including EQ-5D utility instrument and two visual analogue scales (VAS) measuring overall health and fatigue in three French centers (Paris, Lille and Montpellier). Answers were analyzed in mixed models (taking into account the clustering effects of centers and physicians).

**Results:**

Five hundreds and five patients were enrolled: 52% males; the mean age was 54; 41% had BMI>25; 64% had genotype 1; 36% were at the stage of severe fibrosis (F3-F4); 38% had severe comorbidities other than liver-related. In the univariate analysis, EQ-5D utility was associated with socio-demographic variables as age, place of birth, education, and employment; CHC-related variables as conditions of HCV screening and severity of fibrosis; CHC-unrelated variables as comorbidities other than CHC, being overweight, and psychiatric disorders; feelings about CHC disease as perception of progression, lack of information on CHC and its treatments, and entourage’s feeling. In multivariate analysis, EQ-5D utility was affected by not being in employment (0.72 vs. 0.80), having severe comorbidities other than CHC (0.72 vs. 0.79), being overweight (0.73 vs. 0.78), and feeling worried about CHC progression (0.66 vs. 0.72–0.84). Similar results were found for the VAS.

**Conclusions:**

The presence of severe comorbidities and worrying about CHC progression, but not stage of fibrosis, seem to alter significantly EQ-5D health utility in CHC French patients.

## Introduction

The impact of chronic hepatitis C (CHC) infection on health-related quality of life (HRQoL) has received increasing interest over the past ten years [1,2]. HRQoL has been especially shown to be impaired in patients with CHC [3–6]. With the advent of highly effective, and well tolerated, new direct-acting antivirals (DAAs), clinicians and patients emphasize that all those with HCV should receive these new treatments, not only to decrease HCV morbidity and mortality and/or HCV transmission, but also to improve patients' HRQoL. Indeed, higher HRQoL has been found after a sustained virological response [7,8]. The hypothesis is that, because of the impact of these treatments on HRQoL, and despite their high costs, they could be cost-effective even in those patients who are at the early stages of the disease. However, few studies have measured HRQoL [7,9,10].

HRQoL may be affected in a subgroup of HCV-infected patients although not all of them. A recent study conducted in France, the UK, and Germany generated a utility value set for CHC patients, stratified by stage of liver disease [8]. Patient characteristics such as male gender [11,12], being unemployed, being an intravenous drug user, comorbidities, a liver disease stage above F2 [4,13–15], genotype 3, and not having achieved sustained virological response (SVR), decreased HRQoL. Associations were also found between HRQoL and knowledge of diagnosis [16,17] and or being naïve to treatment or not (in relation to adverse events of previous treatment) [18–20].

Beyond the level of disease severity, HRQoL for CHC patients may be impaired by the difficulty of accepting the disease and its social impacts. These non-clinical aspects had not been widely assessed. The availability of new DAAs may change not only the HRQoL of CHC patients, but also their perception of the disease.

The objective of the present study was to evaluate the HRQoL and their determinants in untreated patients with CHC, before the initiation of new DAAs in France, in a period during which patients were aware of DAAs availability.

## Methods

### Study design and participants

The French ethics committee, Commission nationale de l'informatique et des libertés de France (CNIL) approved by written the study (N/Ref.: MMS/FLR/AR146546). A cross-sectional study was conducted between January 2014 and January 2015 in three reference centers (Paris, Lille and Montpellier). Patients with CHC were eligible for this study if they were 18–70 years old, had not received a liver transplant, and were not receiving treatment for CHC. All patients meeting the inclusion criteria were invited by their physician to participate. The individual in this manuscript has given written informed consent (as outlined in PLOS consent form) to publish these case details.

All participants completed a questionnaire administered face to face by a clinical research fellow, except at Lille, where it was self-administered. The questionnaire proposed 50 questions including measures of HRQoL and patients’ characteristics: comorbidities, circumstances of HCV discovery, CHC clinical history and its evolution, information and perception of CHC (using four-point Likert scales [21]), impact of CHC on daily life including dependence on alcohol (according to a CAGE (Cut-down, Annoyed, Guilty, Eye-opener) questionnaire [22]), and positioning in relation to HCV treatments. Fibrosis stage and genotype were obtained from medical records.

### Measure of quality of life

HRQoL was assessed using EQ-5D, for which a time trade-off (TTO) French utility value set is available [23]. This questionnaire contains five generic questions on mobility, self-care, habitual activities, pain/discomfort and anxiety/depression that were declined on three levels (EQ-5D-3L): no problems, some problems, and extreme problems.

HRQoL was also evaluated through two visual analogue scales (VAS), a vertical line of 100 mm: one assessing overall health [24] defined between 0 (worst) and 100 (best imaginable health), and one assessing overall fatigue [25] defined between 0 (none) and 100 (worst fatigue). The patient placed a mark between these two extremities according to his overall health and his level of fatigue felt the day of the interview.

### Statistical analysis

Chi-square tests were used to compare patients’ characteristics according to their stage of fibrosis (F0-F1, F2 and F3-F4). HRQoL was evaluated through the estimation of mean EQ5D utility index [23], and mean VAS values on general health [24] and fatigue [25]. Determinants of the EQ-5D utility index, overall health, and overall fatigue, were analyzed with mixed models taking into account the cluster effect (hepatology centers and physicians). Variables associated with the outcome in the univariate analysis (p<0.20) were introduced in the multivariate analysis. To avoid the inclusion of highly correlated variables in the multivariate analysis, we performed a principal component analysis on all 12 subjective Likert scales on knowledge, attitude, belief, and perception. Accordingly, four variables added little information and were removed: perceived future progression of CHC, severity of CHC disease, knowledge about the availability of new treatments, and knowledge about the chances of healing using older treatments. Next, a step-by-step backward elimination procedure with a significance threshold of 0.05 was used to identify the variables independently associated with each outcome studied, while four socio-demographic variables (gender, age, place of birth, education) were forced in all multivariate models. Data were analyzed using SAS version 9.4.

## Results

### Demographic characteristics and clinical data

A total of 505 HCV-mono-infected patients were enrolled in the study (Table 1); 481 had complete records. The detailed socio-demographic and clinical characteristics of patients are presented according to fibrosis stage. Forty-one percent of patients had minimal fibrosis (F0-F1), whereas 35% were at an advanced stage F3-F4. Overall, 52% were men; the majority of patients were over 50 years old (72%), born in France (74%), had a bachelor's degree level of education (54%), were in employment (55%), and had children (75%). The distribution of gender, age, education and parenthood varied according to fibrosis stage. In particular, women represented 58% of F0-F1, 46% of F2 and 36% of F3-F4 (*p*<0.0001).

**Table 1:** Patient characteristics, n (%).

Variable	Class	Total	F0-F1	F2	F3-F4	*P* value
Gender	Male	252 (52.4)	81 (41.5)	62 (53.5)	109 (64.1)	< .0001
	Female	229 (47.6)	114 (58.5)	54 (46.5)	61 (35.9)	
Age	18–49	133 (27.7)	75 (38.5)	22 (19.0)	36 (21.2)	0.006
	50–54	102 (21.2)	36 (18.5)	25 (21.6)	41 (24.1)	
	55–59	104 (21.6)	39 (20.0)	26 (22.4)	39 (22.9)	
	60–64	76 (15.8)	23 (11.8)	22 (19.0)	31 (18.2)	
	65–70	66 (13.7)	22 (11.3)	21 (18.1)	23 (13.5)	
Place of birth	France	354 (73.6)	136 (69.7)	88 (75.9)	130 (76.5)	0.284
	Abroad	127 (26.4)	59 (30.3)	28 (24.1)	40 (23.5)	
Education	No bachelor level	223 (46.4)	75 (38.5)	58 (50.0)	90 (52.9)	0.015
	Bachelor level	258 (53.6)	120 (61.5)	58 (50.0)	80 (47.1)	
Work	Yes	263 (54.7)	111 (56.9)	69 (59.5)	83 (48.8)	0.148
	No	218 (45.3)	84 (43.1)	47 (40.5)	87 (51.2)	
Children	Yes	361 (75.1)	134 (68.7)	96 (82.8)	131 (77.1)	0.016
	No	120 (24.9)	61 (31.3)	20 (17.2)	39 (22.9)	
Centers	Paris Cochin	221 (45.9)	104 (53.3)	52 (44.8)	65 (38.2)	0.001
	Lille	112 (23.3)	52 (26.7)	26 (22.4)	34 (20.0)	
	Montpellier	148 (30.8)	39 (20.0)	38 (32.8)	71 (41.8)	
Physician	A	28 (5.8)	19 (9.7)	2 (1.7)	7 (4.1)	< .0001
	B	72 (14.9)	31 (15.9)	22 (19.0)	19 (11.2)	
	C	12 (2.5)	2 (1.0)	2 (1.7)	8 (4.7)	
	D	17 (3.5)	7 (3.6)	5 (4.3)	5 (2.9)	
	E	24 (4.9)	15 (7.7)	1 (0.9)	8 (4.7)	
	F	121 (25.2)	54 (27.7)	37 (31.9)	30 (17.7)	
	G	45 (9.4)	21 (10.8)	8 (6.9)	16 (9.4)	
	H	14 (2.9)	7 (3.6)	1 (0.9)	6 (3.5)	
	I	148 (30.8)	39 (20.0)	38 (32.8)	71 (41.8)	
Screening context	Health check-up	259 (54.1)	107 (54.9)	61 (52.6)	91 (54.2)	0.271
	Medical follow-up	40 (8.4)	19 (9.7)	12 (10.3)	9 (5.4)	
	Blood donation	106 (22.1)	46 (23.6)	26 (22.4)	34 (20.2)	
	Persistent fatigue	74 (15.6)	23 (11.8)	17 (14.7)	34 (20.2)	
Genotype	1	311 (64.7)	127 (65.1)	82 (70.7)	102 (60.0)	0.416
	2.3	94 (19.5)	39 (20.0)	17 (14.7)	38 (22.4)	
	4.5.6	76 (15.8)	29 (14.9)	17 (14.7)	30 (17.7)	
Treatment history	Naïve	240 (49.9)	131 (67.2)	51 (44.0)	58 (34.1)	< .0001
	Non-naïve	241 (50.1)	64 (32.8)	65 (56.0)	112 (65.9)	
Severe comorbidities^a^	Yes	180 (37.4)	67 (34.4)	42 (36.2)	71 (41.8)	0.329
	No	301 (62.6)	128 (65.6)	74 (63.8)	99 (58.2)	
Overweight^b^	Yes	200 (41.6)	66 (33.9)	57 (49.1)	77 (45.3)	0.014
	No	281 (58.4)	129 (66.1)	59 (50.9)	93 (54.7)	
Psychiatric disorders	Yes	53 (11.0)	16 (8.2)	14 (12.1)	23 (13.5)	0.247
	No	428 (89.0)	179 (91.8)	102 (88)	147 (86.5)	
Injection or nasal drug use	Yes	201 (41.8)	64 (32.8)	50 (43.1)	87 (51.2)	0.002
	No	280 (58.2)	131 (67.2)	66 (56.9)	83 (48.8)	
Alcohol Use Disorders^c^	Yes	152 (31.6)	41 (21.0)	28 (24.1)	83 (48.8)	< .0001
	No	329 (68.4)	154 (79.0)	88 (75.9)	87 (51.2)	
Perceived progression of CHC between infection and today	Very comforting	56 (12.2)	37 (20.2)	10 (8.9)	9 (5.6)	< .0001
	Rather comforting	250 (54.6)	115 (62.8)	75 (66.4)	60 (37.0)	
	Rather worrying	112 (24.3)	23 (12.6)	22 (19.5)	67 (41.4)	
	Very worrying	40 (8.7)	8 (4.4)	6 (5.3)	26 (16.1)	
Perceived future progression of CHC	Very comforting	96 (20.1)	51 (26.4)	21 (18.3)	24 (14.1)	0.081
	Rather comforting	158 (33.1)	62 (32.1)	39 (33.9)	57 (33.5)	
	Rather worrying	164 (34.3)	56 (29.0)	44 (38.3)	64 (37.7)	
	Very worrying	60 (12.6)	24 (12.5)	11 (9.6)	25 (14.7)	
Level of information^d^	Informed	407 (85.2)	166 (85.6)	96 (83.5)	145 (85.8)	0.845
	Uninformed	71 (14.9)	28 (14.4)	19 (16.5)	24 (14.2)	
Personal research on CHC^e^	Yes	338 (70.3)	142 (72.8)	84 (72.4)	112 (65.9)	0.297
	No	143 (29.7)	53 (27.2)	32 (27.6)	58 (34.1)	
Severity of CHC disease^f^	Not serious	32 (6.7)	17 (8.8)	11 (9.6)	4 (2.4)	0.020
	Rather serious	251 (52.6)	104 (53.6)	64 (55.7)	83 (49.4)	
	Very serious	194 (40.7)	73 (37.6)	40 (34.8)	81 (48.2)	
Knowledge of the availability of new treatments^g^	Very knowledgeable	103 (21.8)	46 (24.0)	31 (27.0)	26 (15.7)	0.119
	Fairly well informed	243 (51.4)	93 (48.4)	53 (46.1)	97 (58.4)	
	Uninformed	127 (26.8)	53 (27.6)	31 (27.0)	43 (25.9)	
Knowledge of chances of healing with older treatments	Yes	295 (62.1)	118 (61.1)	70 (61.4)	107 (63.7)	0.87
	No	180 (37.9)	75 (38.9)	44 (38.6)	61 (36.3)	
Knowledge of chances of healing with new treatments	Yes	341 (71.9)	132 (69.1)	89 (77.4)	120 (71.4)	0.291
	No	133 (28.1)	59 (30.9)	26 (22.6)	48 (28.6)	
Living with CHC in society	Difficult to live in society	261 (56.7)	109 (59.6)	66 (57.4)	86 (53.1)	0.474
	Easy to live in society	199 (43.3)	74 (40.4)	49 (42.6)	76 (46.9)	
Need to talk with people with CHC	Very often	9 (1.9)	3 (1.6)	2 (1.8)	4 (2.5)	0.722
	Often	46 (9.9)	21 (11.2)	10 (8.8)	15 (9.2)	
	Occasionally	141 (30.4)	53 (28.3)	31 (27.2)	57 (35.0)	
	Not at all	268 (57.8)	110 (58.8)	71 (62.3)	87 (53.4)	
Speak freely about CHC with friends	Yes	326 (68.3)	123 (64.1)	75 (65.2)	128 (75.3)	0.051
	No	151 (31.7)	69 (35.9)	40 (34.8)	42 (24.7)	
Entourage uncomfortable since their knowledge of the CHC	Yes, they are uncomfortable	73 (15.4)	24 (12.6)	19 (16.7)	30 (17.9)	0.032
	None is uncomfortable	307 (64.9)	118 (61.8)	72 (63.2)	117 (69.6)	
	No, because nobody knows	93 (19.7)	49 (25.7)	23 (20.2)	21 (12.5)	

N = 481 excluding genotype and stage of fibrosis unknown

^a^Diabetes, non-liver related transplant, psychiatric disorders, drug addiction / alcohol

^b^BMI> 25 kg/m^2^

^c^Patients hospitalized for alcohol dependence or currently receiving substitution treatment

^d^"Uninformed" regroups "rather uninformed" and "very badly informed"

^e^Except the physician’s information

^f^”Not serious” regroups "Not serious at all" and "Rather not matter"

^g^“Uninformed” regroups "Rather uninformed" and "Very badly informed"

Regarding the information about CHC, the majority of patients were in Paris (46%), had been screened for HCV through routine health check-ups (54%), and had a genotype-1 virus (65%). The distribution of centers and physicians, and the treatment history, varied according to fibrosis stage: the majority of F0-F1 and F2 patients were enrolled in Paris (53% and 45%, respectively), whereas the majority of F4 patients were enrolled in Montpellier (42%) (*p* = 0.001); 67% F0-F1 and 34% of F3-F4 were treatment-naïve (*p*<0.0001).

Regarding diseases other than CHC, 37% of patients reported severe comorbidities, 42% were overweight (BMI>25 kg/m^2^), and 11% had psychiatric disorders. Thirty-two percent of patients had alcohol dependence and 42% reported a personal history of injection or nasal drug use. The percentages of patients who were overweight, patients who engaged in drug use, and especially patients with alcohol dependence, increased with fibrosis stage: 21% of F0-F1 vs. 49% of F3-F4 had alcohol dependence (*p*<0.0001).

### Perception of the disease, level of information, and living with CHC

For 67% of patients (Table 1), the perceived progression of CHC between infection and the day of interview was very or rather comforting (83% among F0-F1 vs. 43% among F3-F4, *p*<0.0001). When looking ahead to years to come, 53% perceived CHC progression as very or rather comforting (58% among F0-F1 vs. 48% among F3-F4, *p* = 0.08). By contrast, the disease was perceived as very serious in 41% of patients (38% among F0-F1 vs. 48% among F3-F4, *p* = 0.02). Regarding knowledge of treatments, 73% felt informed about the availability of new treatments, 62% reported having knowledge about the chances of recovery with older treatments (72% with regard to new treatments), without any difference according to stage of fibrosis.

For 57% of patients it was difficult to live with CHC in society. Thirty-two percent of patients said they spoke freely about their CHC disease with their friends (36% among the F0-F1 vs. 25% for F3-F4, *p* = 0.05). Finally, 15% of patients felt that their entourage had been uncomfortable since finding out about their CHC status (13% among the F0-F1 vs. 18% for F3-F4), whereas 20% reported that members of their entourage did not know about their CHC (26% for F0-F1 vs. 12% for F3-F4).

### Estimation of utilities and their determinants

The mean EQ-5D utility was 0.80, while mean VAS values were 68.8 for overall health and 44.7 for overall fatigue. In univariate analyses (Table 2), mean EQ-5D utility decreased with: older age (≥0.80 before 65 years old vs. 0.72 at 65–70 years old); lower levels of education (0.85 in patients with bachelor level vs. 0.75 in those without); being unemployed (0.86 for those working vs. 0.73 for those not working); having children (0.84 in patients without children vs. 0.79 in those with children). Mean EQ-5D utility also varied according to reference center, according to physician, and according to screening context. It also decreased both as the stage of fibrosis advanced (0.83 in F0-F1 and 0.82 in F2 vs. 0.76 in F3-F4) and in the presence of other diseases.

**Table 2:** Determinants of HRQoL: Univariate analysis.

Variable	Class	EQ-5D	*P* value	VAS Health	*P* value	VAS fatigue	*P* value
Gender	Male	0.81	0.197	69.0	0.947	42.9	0.226
	Female	0.79		68.8		46.1	
Age	18–49	0.81	0.028	68.2	0.747	44.8	0.717
	50–54	0.81		68.1		47.2	
	55–59	0.84		69.3		43.7	
	60–64	0.80		71.7		41.2	
	65–70	0.72		67.4		44.5	
Place of birth	France	0.80	0.922	70.2	0.019	45.2	0.339
	Abroad	0.80		65.0		42.3	
Education	No bachelor level	0.75	< .0001	65.7	0.003	47.5	0.038
	Bachelor level	0.85		71.6		42.0	
Work	Yes	0.86	< .0001	72.9	< .0001	41.6	0.017
	No	0.73		64.0		47.9	
Children	Yes	0.79	0.029	68.2	0.202	45.7	0.090
	No	0.84		71.2		40.3	
Centers	Paris	0.86	< .0001	72.3	0.005	39.4	0.002
	Lille	0.75		65.4		49.3	
	Montpellier	0.76		66.2		48.6	
Physician	A	0.83	0.002	66.3	0.173	51.1	0.009
	B	0.73		65.4		47.2	
	C	0.70		64.0		58.5	
	D	0.86		70.0		40.0	
	E	0.87		75.8		30.0	
	F	0.86		72.1		42.8	
	G	0.83		72.2		35.5	
	H	0.91		72.4		32.5	
	I	0.76		66.2		48.6	
Screening context	Health check-up	0.82	0.015	69.2	0.050	42.6	0.076
	Medical follow-up	0.87		76.0		39.2	
	Blood donation	0.75		68.4		46.6	
	Persistent fatigue	0.79		64.5		51.0	
Fibrosis stage	F0-F1	0.83	0.019	73.3	0.002	42.0	0.316
	F2	0.82		66.4		46.4	
	F3-F4	0.76		65.8		45.9	
Genotype	1	0.81	0.576	70.0	0.227	44.2	0.961
	2.3	0.78		67.7		44.9	
	4.5.6	0.80		65.6		45.1	
Treatment-naïve	Naïve	0.81	0.299	70.3	0.153	41.6	0.029
	Non-naïve	0.79		67.5		47.3	
Severe comorbidities^a^	Yes	0.75	< .0001	65.3	0.007	46.9	0.175
	No	0.83		70.9		43.1	
Overweight^b^	Yes	0.77	0.007	68.0	0.432	47.8	0.039
	No	0.82		69.5		42.2	
Psychiatric disorders	Yes	0.73	0.01	59.2	0.001	58.3	0.001
	No	0.81		70.1		42.8	
Injection or nasal drug use	Yes	0.82	0.173	68.3	0.623	45.9	0.354
	No	0.79		69.3		43.4	
Alcohol Use Disorders^c^	Yes	0.79	0.312	68.0	0.563	46.9	0.227
	No	0.81		69.3		43.4	
Perceived progression of CHC between infection and today	Very comforting	0.88	< .0001	80.2	< .0001	31.3	< .0001
	Rather comforting	0.84		71.9		42.2	
	Rather worrying	0.73		61.0		54.3	
	Very worrying	0.66		55.3		51.1	
Perceived future progression of CHC	Very comforting	0.87	< .0001	76.3	< .0001	33.5	< .0001
	Rather comforting	0.83		71.1		40.8	
	Rather worrying	0.79		67.1		47.9	
	Very worrying	0.66		55.5		63.4	
Level of information^d^	Informed	0.82	0.001	70.4	0.001	43.4	0.038
	Uninformed	0.72		59.8		51.3	
Personal research on CHC^e^	Yes	0.81	0.324	68.6	0.683	45.4	0.285
	No	0.79		69.5		42.3	
Severity of CHC disease^f^	Not serious	0.80	0.025	70.1	0.172	36.0	0.131
	Rather serious	0.83		70.5		43.9	
	Very serious	0.77		66.7		46.7	
Knowledge of the availability of new treatments^g^	Very knowledgeable	0.81	0.008	72.9	< .0001	42.4	0.066
	Fairly well informed	0.83		70.9		42.8	
	Uninformed	0.75		61.3		49.7	
Knowledge of chances of healing with older treatments	Yes	0.82	0.005	70.5	0.032	43.8	0.515
	No	0.77		66.2		45.6	
Knowledge of chances of healing with new treatments	Yes	0.82	0.008	70.5	0.007	44.0	0.577
	No	0.76		64.6		45.7	
Living with CHC in society	Difficult to live in society	0.77	0.005	64.8	0.003	51.0	< .0001
	Easy to live in society	0.83		72.0		39.6	
Need to talk with people with CHC	Very often	0.82	0.307	57.5	0.039	51.3	0.005
	Often	0.78		67.3		44.2	
	Occasionally	0.78		65.9		51.2	
	Not at all	0.82		71.1		40.8	
Speak freely about CHC with friends	Yes	0.80	0.506	69.7	0.201	44.2	0.758
	No	0.81		67.0		45.1	
Entourage uncomfortable since their knowledge of the CHC	Yes, they are uncomfortable	0.71	0.001	59.2	< .0001	58.2	< .0001
	Noone is uncomfortable	0.83		72.2		41.2	
	No, because nobody knows	0.80		65.6		44.4	

^a^Diabetes, non-liver related transplant, psychiatric disorders, drug addiction / alcohol

^b^BMI>25 kg/m^2^

^c^Patients hospitalized for alcohol dependence or currently receiving substitution treatment

^d^“Uninformed” groups together “rather uninformed” and “very badly informed”

^e^Except the physician’s information

^f^“Not serious” groups together “not serious at all” and “mostly not serious”

^g^“Uninformed” groups together “rather uninformed” and “very badly informed”

Mean EQ-5D utility decreased along with worse perceptions of CHC progression between infection and the day of enrollment (from 0.88 when very reassuring to 0.66 when very worrying), and of future progression of CHC (from 0.87 when very reassuring to 0.66 when very worrying). Moreover, it also decreased both when patients were not informed about CHC (from 0.82 to 0.72), and with a worse perception of CHC disease (from 0.80 to 0.77). Mean EQ-5D utility also increased with the level of knowledge of the availability of new treatments (from 0.75 to 0.83) and of the chances of healing with new treatments (from 0.76 to 0.82); the utilities were higher when patients were informed. Finally, mean EQ-5D was lower when the perception of life in society with CHC was difficult (0.77 vs. 0.83 when easy), and also when the feeling of the entourage was uncomfortable (0.71 vs. 0.83 when no one was uncomfortable).

Regarding VAS, similar associations were found (Table 2) except that age was not associated either with general health and fatigue, or with the severity of the CHC disease. Moreover, as general health and fatigue deteriorated, the need to talk with people with CHC increased.

Finally, genotype, drug use and alcohol dependence did not affect either mean EQ-5D utilities or mean VAS values for overall health and fatigue.

In multivariate analysis (Table 3), after adjustment on socio-demographic variables (gender, age, place of birth, education) and cluster effects (center and physician), EQ-5D utility remained significantly associated with being unemployed (0.80 for patients working vs. 0.72 for those not working), with the presence of severe comorbidities (0.79 without vs. 0.72 with comorbidities), with being overweight (0.78 without vs. 0.73 with overweight), and with a CHC progression perceived as rather or very worrying (from 0.84 for patients feeling CHC progression was very reassuring, to 0.66 for patients feeling it was very worrying).

**Table 3:** Determinants of HRQoL: Multivariate analysis.

Variable	Class	EQ5D (N = 420)	*P* value	VAS health (N = 416)	*P* value	VAS fatigue (N = 416)	*P* value
Gender	Male	0.78 (0.73–0.82)	0.022	60.9 (56.7–65.1)	0.512	50.1 (42.1–58.2)	0.059
	Female	0.74 (0.69–0.78)		59.6 (55.8–63.5)		55.0 (47.5–62.6)	
Age	18–49	0.74 (0.70–0.79)	0.617	57.8 (53.4–62.2)	0.155	53.4 (45.3–61.6)	0.573
	50–54	0.74 (0.69–0.80)		57.7 (53.1–62.3)		56.2 (47.9–64.6)	
	55–59	0.78 (0.73–0.83)		58.6 (53.7–63.4)		52.5 (43.7–61.3)	
	60–64	0.76 (0.71–0.82)		62.9 (57.4–68.5)		49.8 (40.3–59.3)	
	65–70	0.75 (0.69–0.81)		64.3 (58.3–70.4)		50.9 (41.1–60.7)	
Place of birth	France	0.75 (0.71–0.79)	0.528	62.8 (59.2–66.3)	0.017	53.9 (46.5–61.4)	0.350
	Abroad	0.76 (0.71–0.81)		57.8 (53.2–62.4)		51.2 (42.8–59.6)	
Education	No bachelor level	0.74 (0.69–0.78)	0.041	59.9 (55.9–63.9)	0.689	53.3 (45.5–61.1)	0.567
	Bachelor level	0.78 (0.73–0.82)		60.7 (56.6–64.7)		51.8 (44.0–59.7)	
Work	Yes	0.80 (0.75–0.84)	0.001	63.8 (59.5–68.0)	0.001		
	No	0.72 (0.67–0.76)		56.8 (52.9–60.7)			
Fibrosis stage	F0-F1			62.7 (58.2–67.1)	0.039		
	F2			56.9 (52.3–61.5)			
	F3-F4			61.2 (57.0–65.4)			
Severe comorbidities^a^	Yes	0.72 (0.67–0.77)	< .0001	57.7 (53.7–61.8)	0.008		
	No	0.79 (0.75–0.84)		62.8 (58.9–66.7)			
Overweight^b^	Yes	0.73 (0.69–0.78)	0.017			55.3 (47.6–63.1)	0.031
	No	0.78 (0.74–0.82)				49.8 (41.9–57.7)	
Psychiatric disorders	Yes			57.0 (51.2–62.7)	0.023	58.3 (48.6–67.9)	0.004
	No			63.6 (60.8–66.4)		46.9 (40.1–53.7)	
Perceived progression of CHC between infection and today	Very comforting	0.84 (0.78–0.90)	< .0001	71.2 (65.2–77.1)	< .0001	41.6 (31.4–51.8)	< .0001
	Rather comforting	0.82 (0.77–0.86)		64.8 (61.2–68.4)		50.5 (43.0–58.0)	
	Rather worrying	0.72 (0.67–0.77)		54.2 (49.6–58.7)		61.1 (53.0–69.3)	
	Very worrying	0.66 (0.59–0.73)		51.0 (44.4–57.5)		57.1 (46.6–67.6)	
Need to talk with people with CHC	Very often					57.4 (38.6–76.1)	0.027
	Often					47.2 (37.9–56.6)	
	Occasionally					56.7 (49.7–63.7)	
	Not at all					49.1 (42.9–55.3)	
Entourage uncomfortable since their knowledge of CHC	Yes, they are uncomfortable			57.1 (51.8–62.3)	0.003	59.5 (50.4–68.7)	0.001
	Noone is uncomfortable			64.7 (61.0–68.3)		46.7 (39.2–54.1)	
	No, because nobody knows			59.1 (54.2–64.0)		51.5 (42.6–60.4)	

^a^Diabetes, non-liver related transplant, psychiatric disorders, drug addiction / alcohol

^b^BMI>25 kg/m^2^

Regarding VAS for health and fatigue, similar results were found, except that VAS deteriorated significantly in the presence of psychiatric disorders and for patients feeling that their entourage felt uncomfortable since finding out about their CHC status. Moreover, general health was significantly lower for patients in F2 (56.9) compared to those in F0-F1 (62.7) and F3-F4 (61.2).

## Discussion

This is the first study conducted in France to assess QoL in a large sample of patients affected by CHC. We found that EQ-5D utility values, as well as overall health and fatigue assessed by VAS, were mainly influenced by socio-demographic characteristics (unemployment), by comorbidities (including overweight), and by individual perceptions about CHC progression, rather than by the clinical characteristics of CHC (fibrosis stage, genotype, treatment-naïve status).

Although the stage of fibrosis significantly affects EQ-5D in univariate analysis, it was not found to be an independent factor of HRQoL. Similarly, Hsu *et al* [17,18] did not find an association between HRQoL and the stage of fibrosis, and emphasized the greater impact of socio-demographic variables on HRQoL.

It was not surprising to find that unemployment, overweight, psychiatric disorders, and comorbidities decrease HRQoL. Unemployment was also found to be significantly associated with decreased HRQoL by Pol *et al* [13]. Multiple studies found an association between each of those comorbidities and decreased HRQoL [26–28]. In our study, over a third of patients reported comorbidities such as diabetes, arterial hypertension, chronic kidney disease, or hemophilia. During the interview, patients reporting comorbidities were systematically asked to directly compare the severity of CHC with that of other comorbidities (N = 222). Forty-four percent felt that CHC was more severe than their other comorbidities, 36% felt CHC was as severe, but 20% felt CHC was less severe than other comorbidities (not shown). These results suggest that the eradication of HCV after treatment will not necessarily improve the HRQoL of those patients with comorbidities and emphasize a need for active therapeutic interventions regarding these comorbidities.

Patients perception on CHC disease was found to be associated with HRQoL. This perception was principally related to the stage of fibrosis. Patients who saw their disease progress to F3-F4 at the moment of this study were obviously more worried than those at F0-F2. However, despite minimal fibrosis, F0-F1 patients were also found to be worried (17% seeing CHC disease as rather or very worrying). Indeed, as stated by others, HRQoL also decreases along with patients' anxiety regarding the evolution of their disease [29] which may be unrelated to fibrosis stage. For example, we found that HRQoL was also impacted by the feeling of the entourage. In a large proportion of patients, the entourage was unware of the CHC status or, when knowing it, felt uncomfortable. In these cases, these feelings may contribute to a sense of isolation on the part of the patient; again regardless to the fibrosis stage.

Our study has some limitations. First, it was conducted in only three reference centers. These were deliberately chosen to yield a geographically comprehensive representation of patients in France. Despite this limitation, patients’ characteristics were representative of CHC patients in France, with a majority of men, an average age of around 54, and two-thirds of patients with genotype 1 [30]. Second, we used the EQ-5D instrument to assess the self-reported HRQoL of patients because it is a commonly used generic measure of health that is recommended for analyzing cost-effectiveness [31,32]. However, it was reported not to be very sensitive to variations in HRQoL [32–33]. Therefore we added more sensitive measurement instruments with two visual analogue scales on overall health and fatigue. As in previous studies, the main results based on EQ-5D utility were similar to those obtained with the more sensitive VAS scores. Finally, our study took place over the year 2014; one may consider that the patients knowledge of new DAA at that time where less than today. Thus, a study conducted today may in particular result into patients with higher quality of lives.

In conclusion, factors such as the perceived progression of CHC, the need among patients to share the experience of their disease by talking with other CHC sufferers, and a patient's entourage being uncomfortable in the knowledge of their CHC diagnosis, significantly impact HRQoL, but not the fibrosis stage. These variables should be considered when providing care to HCV patients and deciding which subgroup of patients should be treated. The presence of comorbidities, frequent in patients with HCV disease, was also associated with HRQoL. This may imply that the eradication of HCV does not necessarily improve the HRQoL of patients who continue to live with other diseases.
